# Hepatitis C virus (HCV) genotypes distribution: an epidemiological up-date in Europe

**DOI:** 10.1186/s13027-016-0099-0

**Published:** 2016-10-12

**Authors:** Arnolfo Petruzziello, Samantha Marigliano, Giovanna Loquercio, Carmela Cacciapuoti

**Affiliations:** Virology and Molecular Biology Unit “V. Tridente”, Istituto Nazionale Tumori - Fondazione “G. Pascale”, IRCCS Italia, Via Mariano Semmola, 80131 Naples, Italy

**Keywords:** HCV genotype, Epidemiology, Hepatitis C virus, HCV prevalence, Viraemia, HCV infections

## Abstract

Hepatitis C virus (HCV) infection is a major public health burden in Europe, causing an increasing level of liver-related morbidity and mortality, characterized by several regional variations in the genotypes distribution.

A comprehensive review of the literature from 2000 to 2015 was used to gather country-specific data on prevalence and genotype distribution of HCV infection in 33 European countries (about 80 % of the European population), grouped in three geographical areas (Western, Eastern and Central Europe), as defined by the Global Burden of Diseases project (GBD).

The estimated prevalence of HCV in Europe is 1.7 % showing a decrease than previously reported (− 0.6 %) and accounting over 13 million of estimated cases. The lowest prevalence (0.9 %) is reported from Western Europe (except for some rural areas of Southern Italy and Greece) and the highest (3.1 %) from Central Europe, especially Romania and Russia. The average HCV viraemic rate is 72.4 %, with a population of almost 10 million of HCV RNA positive patients.

Genotype distribution does not show high variability among the three macro-areas studied, ranging between 70.0 % (Central Europe), 68.1 % (Eastern Europe) and 55.1 % (Western Europe) for genotype 1, 29.0 % (Western Europe), 26.6 % (Eastern Europe) and 21.0 % (Central Europe) for genotype 3. Genotype 2 seems, instead, to have a major prevalence in the Western Europe (8.9 %), if compared to Eastern (4.3 %) or Central (3.2 %), whereas genotype 4 is present especially in Central and Western area (4.9 % and 5.8 %, respectively).

Despite the eradication of transmission by blood products, HCV infection continues to be one of the leading blood-borne infections in Europe. The aim of this review is, therefore, to provide an update on the epidemiology of HCV infection across Europe, and to foster the discussion about eventual potential strategies to eradicate it.

## Background

Hepatitis C virus (HCV) is one of the major globally prevalent pathogen and one of the main leading cause of death and morbidity also in Europe [1–3]. According to some estimates, about 3–4 million people are newly infected every year worldwide, and over 350,000 patients yearly die due to HCV-related disorders [4]. The last estimates of disease burden showed an increase in seroprevalence over the last 15 years to 2.8 %, equating to >185 million infections worldwide [5].

The severity of HCV infection is essentially due to its long term hepatic and extrahepatic consequences [6, 7]. Persistent HCV infection is generally associated with the development of liver cirrhosis, hepatocellular carcinoma (HCC), liver failure, and death [8], and a significant portion of liver transplantation in Europe is attributable to disorders related to Chronic Hepatitis C (CHC) [9]. The limited effectiveness of treatments available until a couple of years ago have led HCV-associated mortality to exceed that due to the human immunodeficiency virus (HIV) in developed countries.

The incidence of complications of CHC will not decline over the next 10 years despite improved efficacy of antiviral therapy because most patients with CHC remain undiagnosed [10]. In addition, the clinical impact of some extrahepatic disorders, leading to renal, cardiac and cerebrovascular outcomes associated with cryoglobulinemia and diabetes, has been emphasized only recently [4, 5, 11, 12] and traditionally neglected in cost-effectiveness analyses.

Although a recent meta-analysis indicates the global incidence rate of HCV infection decreasing [13], mathematic models show that deaths from liver disease secondary to HCV infection will continue to increase over the next 20 years [14, 15]. This means that, although many data suggest that HCV infection could be eliminated in the next 15–20 years with focused strategies to cure current infections and prevent new infections [16, 17], a good understanding of HCV epidemiology should be required to develop strategies to eradicate HCV.

The epidemiological status of HCV infection in Europe is continuously evolving and may vary significantly region by region. In the last years several studies have reported level prevalence estimates of HCV infections in Europe, but always considering a limited number of countries or specific risk groups [18–23]. A more recent analysis, instead, estimates a global and also European HCV prevalence, but provides only regional estimates [3]. In all these cases, however, studies were focused only on the presence of HCV antibodies that generally overestimates the disease burden because they include also patients healed spontaneously or through treatments. So, although antibodies to HCV (anti-HCV) are actually the most commonly available marker of HCV infection and often used both to estimate the prevalence of anti-HCV in population-based studies and to compare HCV infection levels globally, the most important indicator of HCV diffusion seems to be its classification into different genetic variants.

HCV exhibits an extraordinarily high degree of genetic diversity [24]. Its strains are classified into seven recognized genotypes on the basis of phylogenetic and sequence analyses of whole viral genomes [25, 26]. HCV strains, belonging to different genotypes, differ at 30–35 % of nucleotide sites. Within each genotype, HCV is further classified into 67 confirmed and 20 provisional subtypes. Strains that belong to the same subtype differ at <15 % of nucleotide sites [27].

Since, actually, the duration of treatment and the need for adjuvant interferon and ribavirin with the new direct-acting antiviral (DAA) therapies still remain dependent in part on HCV genotype and subtype, it is clear how a better knowledge of the epidemiology of HCV and of the distribution of its genotypes could substantially contribute to an effective control of this troubling pandemic especially by focusing screening strategies on patients at risk of disease progression, in order to get them into earlier treatment.

Because epidemiological data are the basis for the development of preventive strategies able to eradicate HCV infection, the aim of this study is to systematically up-date and review HCV epidemiology throughout Europe to foster the development of country-specific screening programs and an international HCV surveillance program.

## Methods

A comprehensive review of the literature from 2000 to 2015 was used to gather country-specific data on prevalence, number of diagnosed individuals and genotype distribution. References were identified through two sources: indexed journals and non-indexed sources. Indexed articles were found by searching Pub Med and regional databases using the following terms: “[Country Name] and [hepatitis c or HCV] and [prevalence]” or [genotypes] or [viraemia]”. Furthermore, references cited within the articles were used.

Regions included in the analysis were those defined by the Global Burden of Diseases, Injuries, and Risk Factors 2010 (GBD) study. This study defined in Europe three regions (Western, Eastern and Central) that were “epidemiologically homogenous as possible so that information from detailed studies in one country can plausibly be extrapolated to other countries in the region to create burden estimates that are useful to individual countries in planning for health sector activities” [28–30].

The average HCV prevalence and viraemic rate for each region were calculated by dividing the sum of data reported from each country to the total number of countries within the region.

Article titles and abstracts were reviewed for relevance and the following data were extracted from full articles or abstracts:anti-HCV prevalence, viraemic prevalence, viraemic rate and genotypes distribution.

Studies in non-representative populations (e.g., people who inject drugs (PWID’s), haemophiliacs, minority ethnic groups, blood donors, etc.) or with a sample size of less than 1000 and studies published prior to 2000 or not in English were excluded from the analysis.

Five hundred twenty nine articles were selected from 33 countries based on relevance. In addition, non-indexed sources were identified through searches of individual country’s Ministry of Health’s websites and international health agency reports. If articles contained the same patient cohort then this cohort was only counted once. No representative data were available from six countries (Albania, Bosnia and Herzegovina, Estonia, Iceland, Macedonia and Montenegro).

Because the first- and second generation immunoassay tests may provide false-positive results, which can overestimate the total infected population, care was taken to use only studies that used the latest generation tests to estimate the country’s prevalence.

In the majority of studies HCV cases were classified at the genotype level, but not at the subtype level, so we decided to use only genotype classification using as general method that proposed by Simmonds et al. [25]. In case of one or more genotypes identified in the same patient, we classified it as “mixed”. We did not include genotype 7 in the analysis.

## General epidemiology of Hcv in Europe

The GBD subdivides Europe into 3 main areas: Central, Eastern and Western. The collected data were segmented by country according to prevalence, HCV genotype distribution and viraemic rate. The European Center for Disease Prevention and Control (ECDC) provided incidence rates for the European countries and their estimates were used for newly diagnosed populations because most countries did not distinguish between chronic and acute cases of HCV infection.

Surveillance systems also vary widely between and within countries in Europe. A publication by ECDC documented 38 different surveillance systems in 27 countries; six countries had more than one system [22]. Surveillance systems, besides, vary by structure, reporting practices, data collection methods and case definitions used [31]. For these reasons, caution should be exercised when comparing case reporting data across countries.

The estimated prevalence of HCV of the whole continent is 1.7 %, ranging from 3.1 % in Eastern Europe to 0.9 % in Western Europe, accounting over 13 millions of estimated cases. The average HCV viraemic rate is 71.3 %, with a population of almost 10 million of HCV RNA positive patients (Table 1).

**Table 1:** HCV Seroprevalence and viraemic rate in Europe

Regions	Anti - HCV Prevalence (%)	Viraemic Rate (%)
Europe, Central	1.2	73.3
Europe, Eastern	3.1	69.6
Europe, Western	0.9	71.0
Total Europe	1.7	71.3

The predominant genotype is genotype 1 (G1) (64.4 %), followed by genotype 3 (G3) (25.5 %), 2 (G2) (5.5 %) and 4 (G4) (3.7 %). Only small percentages of genotype 5 (G5), genotype 6 (G6) and mixed or not further classified genotypes are reported (Table 2).

**Table 2:** Prevalence of HCV genotypes in Europe

Continents	G1 (%)	G2 (%)	G3 (%)	G4 (%)	G5 (%)	G6 (%)	Mixed
Central Europe	70.0	3.2	21.0	4.9	-	0.1	0.8
Eastern Europe	68.1	4.3	26.6	0.5	-	-	0.5
Western Europe	55.1	8.9	29.0	5.8	0.2	0.1	0.8
Total Europe	64.4	5.5	25.5	3.7	0.1	0.1	0.7

Genotype distribution does not show high variability among the three macro-areas studied, ranging between 70.0 % (Central Europe), 68.1 % (Eastern Europe) and 55.1 % (Western Europe) for G1, 29.0 % (Western Europe), 26.6 % (Eastern Europe) and 21.0 % (Central Europe) for G3.

G2 seems to have a major prevalence in the Western Europe (8.9 %), if compared to Eastern (4.3 %) or Central (3.2 %), whereas G4 is present especially in Central and Western area (4.9 % and 5.8, respectively).

Only few cases of G5 and G6 are reported and mainly from Western area (Table 2).

### Central Europe

This large area, including countries like Albania, Bulgaria, Bosnia and Herzegovina, Czech Republic, Croatia, Hungary, Macedonia, Montenegro, Poland, Romania, Serbia, Slovakia and Slovenia, shows a prevalence of HCV infection of 1.2 %, varying between 3.2 % in Romania and 0.5 % in Serbia and an average viraemic rate estimated at 73.3 % (Table 3). We did not found representative data concerning the HCV prevalence from published studies in Albania, Bosnia and Herzegovina, Macedonia and Montenegro.

**Table 3:** HCV prevalence/infected population in Central Europe (adjusted for the adult population)

Region/Country	Number of studies	Anti-HCV prevalence (%)	Anti-HCV infected (thousands)	Viraemic rate (%)
﻿﻿Central Europe	31	1.2	1188.7	73.3
Bulgaria	3	1.1	67	-
Croatia	1	0.9	38.2	-
Czech Republic	1	0.7	60	70.0
Hungary	6	0.8	68	84.6
Poland	10	0.9	279	70.0
Romania	5	3.2	575	91.3
Serbia	1	0.5	35.5	-
Slovakia	1	1.4	66	49.2
Slovenia	3	-	-	74.6

**Fig. 1:**
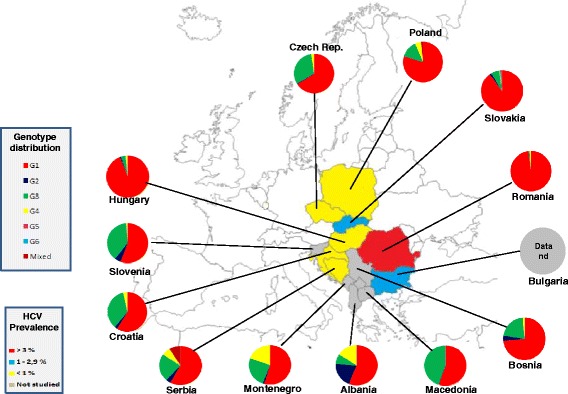
Genotype distribution in Central Europe

The predominant genotypes in this area is G1 (70.0 %), followed by G3 (21.0 %), G4 (4.9 %) and G2 (3.2 %). Only a small percentage of mixed genotypes and G6 has been found, whereas no G5 cases are reported (Fig. 1). In Romania, Hungary and Slovakia, G1 is almost the only genotype found (98.0 , 94.1 and 89.9 %, respectively). A considerable percentage of G3 was described in Macedonia (44.6 %), Slovenia (37.8 %) and Croatia (35.6 %), while a significant prevalence of G2 was described only in Albania (20.0 %) and of G4 in Montenegro (19.6 %) and Albania (16.0 %).

No genotypes distribution data are available from Bulgaria.

### Eastern Europe

The prevalence of HCV infection in this zone, including Belarus, Estonia, Lithuania, Latvia, Moldova, Russia and Ukraine, is 3.1 %, ranging between 4.5 % in Moldova and 1.3 % in Belarus, with a viraemic rate estimated at 69.6 % (Table 4). No adult HCV prevalence and/or viraemic data are available from Estonia.

**Table 4:** HCV prevalence/infected population in Eastern Europe (adjusted for the adult population)

Region/Country	Number of studies	Anti-HCV prevalence (%)	Anti-HCV infected (thousands)	Viraemic rate (%)
Eastern Europe	20	3.1	6682	69.6
Belarus	2	1.3	100	69.0
Latvia	3	2.9	73	71.4
Lithuania	2	2.4	42	-
Moldova	2	4.5	130	-
Russia	8	4.1	4932	-
Ukraine	1	3.6	1385	-

**Table 5:** HCV prevalence/infected population in Western Europe (adjusted for the adult population)

Region/Country	Number of studies	Anti-HCV prevalence (%)	Anti-HCV infected (thousands)	Viraemic rate (%)
Western Europe	478	0.9	3339	71.0
Austria	26	0.5	36	73.4
Belgium	15	0.9	86	80.0
Cyprus	2	0.6	5	71.4
Denmark	8	0.7	33	62.2
Finland	4	0.7	31	-
France	104	0.6	197	65.0
Germany	56	0.6	401	66.7
Greece	25	1.9	178	-
Ireland	7	1.1	40	75.0
Italy	124	2.0	1048	73.3
Luxembourg	1	0.9	4	-
The Netherlands	1	0.2	31	-
Norway	5	0.7	29	79.5
Portugal	4	1.8	164	-
Spain	51	1.7	688	68.6
Sweden	18	0.7	53	77.0
Switzerland	12	1.5	105	-
United Kingdom	15	0.6	210	68.5

**Fig. 2:**
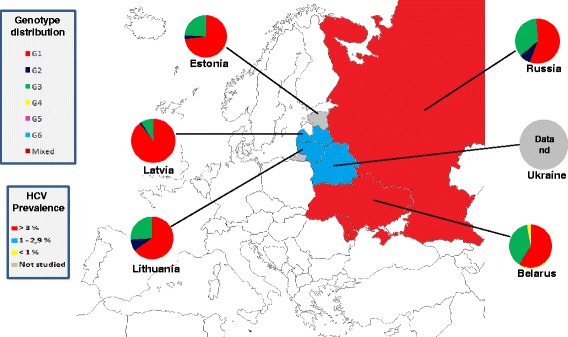
Genotype distribution in Eastern Europe

The predominant genotypes in this area is G1 (68.1 %), followed by G3 (26.6 %) and G2 (4.3 %). Only a small percentage of mixed genotypes and G4 (0.5 %) are reported, whereas no G5 and G6 cases has been described (Fig. 2).

Only in Latvia G1 is the dominant genetic variant (89.2 %). A considerable percentage of G3 was described in Belarus (38.5 %) and Russia (35.1 %).

No genotypes distribution data are available from Moldova and Ukraine.

### Western Europe

The countries studied in this area were Austria, Belgium, Cyprus, Denmark, Finland, France, Germany, Greece, Iceland, Ireland, Italy, Luxembourg, The Netherlands, Norway, Portugal, Spain, Sweden, Switzerland and United Kingdom.

The prevalence of HCV in the general population of this area is 0.9 %, ranging between 2.0 % in Italy and 0.2 % in The Netherlands, with a viraemic rate estimated at 71.0 % (Table 5). No representative data concerning the HCV prevalence from published studies were found from Iceland.

**Fig. 3:**
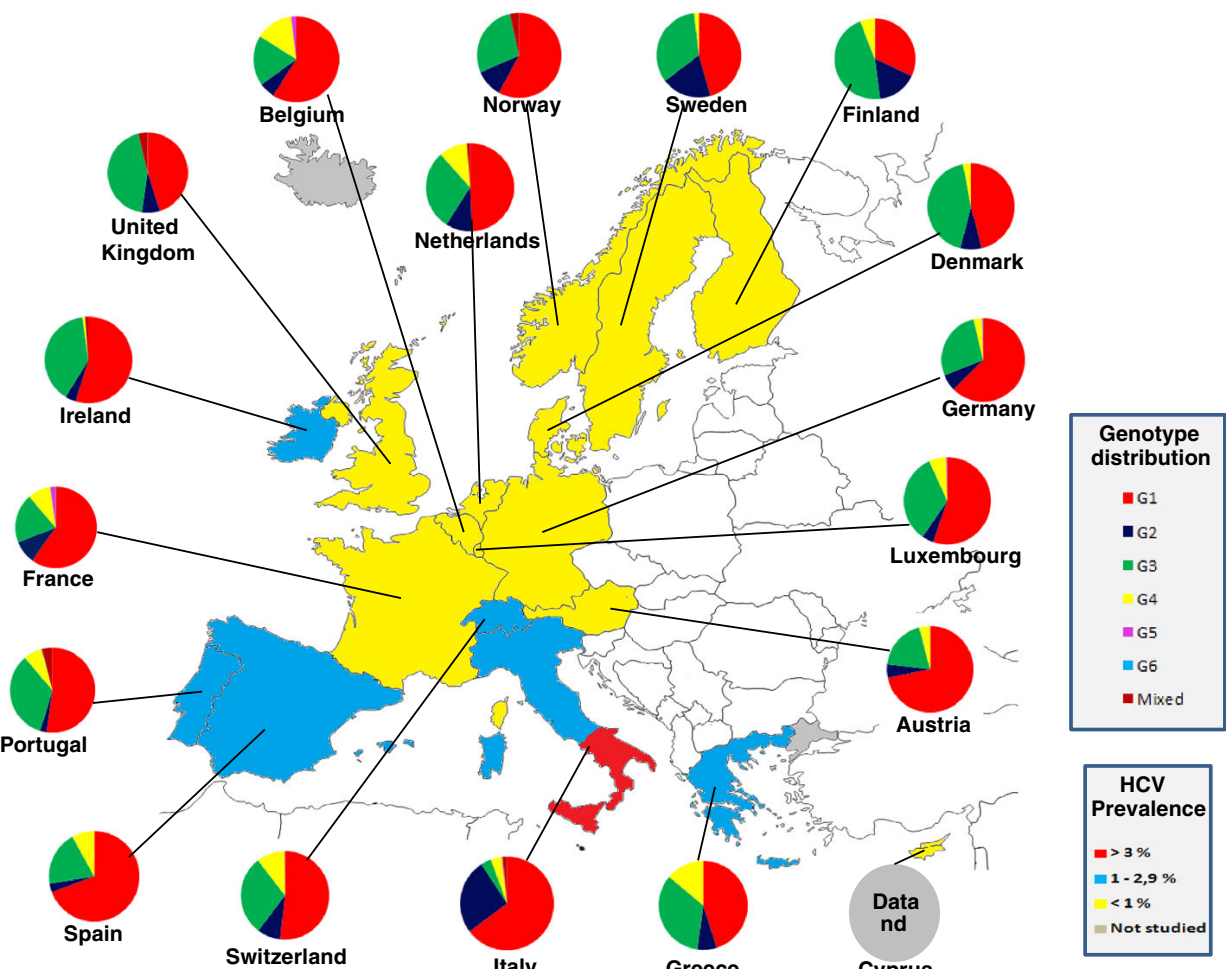
Genotype distribution in Western Europe

The predominant genotypes is G1 (55.1 %), followed by G3 (29.0 %), G2 (8.9 %) and G4 (5.8 %), whereas only small percentages of G5, G6 and mixed genotypes are reported (Fig. 3). In Austria, Spain, Germany and Italy G1 is over the sixty percent of all the genotypes found. A considerable percentage of G3 was described in some of the countries of the Northern Europe, as Finland (46.0 %), United Kingdom (43.8 %), Denmark (43.0 %), whereas only Italy shows a significant percentage of G2 (26.0 %).

No genotypes distribution data are available from Cyprus.

## Discussion and conclusions

Hepatitis C virus (HCV) infection is one of the major public health burden in Europe, causing an increasing level of liver-related morbidity and mortality due to the disease progression [32–38].

The HCV disease paradigm varies by country based on historical and present risk factors, screening programs and treatment rates. Individual countries must consider appropriate country-specific prevention, diagnosis and treatment strategies to reduce the disease burden represented by HCV. Unfortunately, in many countries, there is a lack of robust epidemiological data upon which to base these strategies. Many studies have examined regional HCV infection rates [39–42], but they have typically focused on quantifying the anti-HCVprevalence with no attention to HCV genotypes distribution.

Here we have provided a comprehensive review of HCV epidemiology studies throughout Europe between 2000 and 2015 with a special care to not base our study only on available data but instead trying to analyze only the relevant data. In fact, although HCV prevalence among blood donors, available in many countries, represents surely an attractive data source for the large sample size, all the studies concerning only this subgroup were excluded because this population, corresponding to healthy screened adults, is not representative of the total population. For the same reason, on the contrary, numerous high risk populations studies (e.g. PWIDs, haemodialysis patients, cancer patients, etc.) were not considered too. Finally, all the studies published prior to 2000 were excluded considering the global epidemiological changes that HCV infections has had in the latest twenty years [43–45].

Studying 33 countries (9 in Central Europe, 6 in Eastern Europe, and18 in Western Europe), HCV prevalence in Europe is estimated at 1.7 % (over 10 million of HCV infected adults). Our data suggest that the lowest HCV prevalence estimates are from Western European countries (0.9 %), while the highest from Eastern Europe (3.1 %), even if these percentages should probably be adjusted in the future given the limited evidentiary support, especially from some countries in Central and Eastern European bloc. The main shortcomings of majority of studies from these areas reside in the fact that data are often based on surveys conducted in selected groups [32–34]. Furthermore, many studies are outdated and have failed to take into consideration the influence of some recent drivers such as migratory movements, including those regarding war refugees and illegal human trafficking.

No adult HCV prevalence studies were available from 6 countries (1 both in Central and Western Europe and 4 in Eastern Europe), but in order of their contribution, the studied countries account for over the 80 % of total European population.

Our analysis shows that the prevalence and number of HCV infected patients in Europe, if compared to a similar study concerning the period 1990–2005 and covering the geographical area of Europe defined by the WHO (i.e. including the former USSR republics) [5], has decreased from 2.6 % (95 % uncertainty interval [UI]: 2.4–2.9 %) to 1.7 % (95 % UI: 0.9 %–3.1 %) and from 19 to 13 millions. It is interesting to note that the most relevant decrease has been observed in Western Europe (−1.5 %) and Central Europe (− 1.1 %), whereas Eastern Europe countries register a moderate increase (+0.2 %).

By estimating the total number of HCV RNA positive infections, our data show that the global average viraemic rate is at 72.4 % (9.4 millions of HCV RNA positive cases), varying from 73.3 % in Central Europe to 69.6 % in Eastern Europe (Table 1). It is interesting notice that some countries, where it is reported an high anti-HCV prevalence, also have a low viraemic rate. The most interesting example is Poland where it was found an anti-HCV prevalence of 1.9 % with a viraemic rate of 31 % (a viraemic prevalence of 0.6 %) [42]. A more recent study conducted in the same country by using a confirmatory antibody test has showed an anti-HCV prevalence of 0.86 % [46]. This example suggests the need to study viraemic infections since some historically high antibody prevalence estimates may be influenced by the use of low sensitive screening HCV tests.

Although these data seems to indicate a general decrease of HCV infection especially in Western Europe, a recent modelization has estimated how the numbers of HCV- mortality will increase in the next decades [15]. The disease progression model took into account the historical number of HCV infections, the age and gender distribution, the extent and impact of the movers of the HCV viraemic pool (i.e. so-called inputs and outputs, encompassing acute infections progressing to chronicity, migration movements, treatment uptake succeeding into viral eradication and deaths), the progression rates (based on literature data) and the all-cause mortality data gathered from the Human Mortality Database adjusted for incremental increases due to drug abuse and blood transfusion. This model was applied to several major European countries (i.e. Austria, Belgium, England, France, Germany, Spain and others) [47]. According to this model, in the period 2013–2030, the number of decompensated cirrhosis, the prevalence of HCC in the general population and the liver-related morbidity rate will increase in Europe by 80 %, 75 % and 65 %, respectively. The only exception to this dreaded scenario is represented by France, where these parameters will decrease by 80 %, 85 % and 75 % in the same period, probably due to the large use of more potent antivirals.

A low diagnosis rate is obviously a major hurdle to implement strategies to fight the future health burden of HCV. As shown recently [15], countries where is available a centralized registry, such as Austria, France, Germany and others north Europe countries, tend to boost the highest diagnosis rates (up to 80 % for Sweden), while the lowest rates were reported for southern Europe countries, like Portugal (33 %). An useful strategy of screening should consider all patients with a history of exposure via the traditional routes of HCV infection, limiting the number of patients unaware of their infection, even if this approach is not useful to contain the increasing rate of mortality HCV related. For this purpose it would be necessary a more accurate analysis of the distribution of HCV genotypes and of their circulation in Europe, whose lack of data is one of the major health problems in Europe.

Concerning the genotype distribution, G1 accounts for 64.4 % of all HCV infections among adults making it the most common, either in Central Europe (70.0 %) and in Western Europe (55.1 %). Even if here not reported, many data suggest that the subtypes 1a/1b ratio is dependent on patient age and transmission route, with a major prevalence of subtype G1b in older patients and of subtype G1a in PWIDs [48, 49].

G3 is the second most common genotype (25.5 %), ranging from 29.0 % in Western Europe, 26.0 % in Eastern and 21.0 % in Central Europe. This is also one of the most challenging genotypes for therapy, since only sofosbuvir and daclatasvir are licensed for its treatment, and is often associated with faster rates of fibrosis progression and, as G1, higher prevalence of severe steatosis and hepatocellular carcinoma. G3 has been diagnosed more frequently in drug consumers in certain areas especially in West European countries [50, 51].

G2 is the third most frequent genotype with percentages ranging from 8.9 % in Western Europe to 3.2 % in Central Europe [19, 52–54]. It is significantly associated to females, nosocomial infection or dental therapy and is mostly detected in older patients. Higher proportions of G2 were found in Sweden, Finland, Russia and in some of the ex soviet republics, probably in accordance to the Asian genotypes distribution, and in Italy [55, 56], especially in Southern areas [57, 58]. G2 subtype 2c was probably introduced in Italy as a result of population movements during Italian colonialism at the end of the 19th century, and it did not spread there through intravenous drug use [59].

G4, instead, traditionally associated to Central Africa and the Middle East [5, 13, 54] and mainly related to sexual practices, especially in MSM, and in HIV-coinfected patients [19], shows an average European proportion of around 3.7 %, ranging from 5.8 % in Western Europe and 4.9 % in Central Europe and only small percentage in Eastern Europe (0.5 %). A high prevalence of this genotype has been described in Belgium, Greece and The Netherlands (14.0 %, 13.9 % and 10.5 %, respectively), but also in France and Spain (9.2 % and 8.0 %), probably as consequence of three concomitant processes: increase in immigration from Northern and Central Africa, the use of drugs, and the introduction of G4 subtype d viruses into European networks of MSM and injection drug users [60].

Genotypes 5 and 6 were detected in extremely low frequencies and no association with independent epidemiological parameters was found.

It is necessary to clarify that in this study it was not possible to find a significant association between distribution of HCV genotypes and transmission route, since these data were clearly documented only for 25 % of the studies. Furthermore, a clear classification of each genotype in subtypes was found available only for 40 % of the selected studies. This lack of data has avoided a reliable subtypes distribution analysis, and also of “unresolved” or “mixed” infections that were not always clearly separated from double infections (unmistakable co- existence of two or more HCV strains).

In conclusion, HCV epidemiology shows a high variability across Europe, exhibiting a dynamic process influenced by traditional genotypes prevalence and evolving transmission trends. The early-nineties epidemics of GTs 1b and 2, mainly related to nosocomial transmission, have been partially replaced by a scenario of GTs 1a, 3 and 4 where PWIDs and high-risk sexual practices are the main risk factor for HCV transmission [61, 62]. In fact, despite eradication of transmission by blood products, there is still an increase in HCV incidence in some countries, especially in Eastern Europe, probably due to the increase of PWID rate. Furthermore, other factors may also influence epidemiologic trend of HCV infection within the next years and lead to changes in its epidemiology, as the role of past and current immigration, the increase in sex/drug consume-tourism, HCV re-infections rates in IVDA, generation of new (recombinant) GTs, as well as selection of certain GTs by the current DAAs [63–65].

This review is one of the first attempt for the collection of European HCV data to provide reliable information about the current genotype prevalence situation, and it is also a call to join efforts and encourage further observational studies on HCV genotype prevalence at supra- national level to gain reliable knowledge on the epidemiology of HCV infection. Stronger national and international efforts, including a more massive collection of data especially on risk groups and the institution of a central register to monitor the national HCV diagnosis rates, could surely introduce an appropriate strategyto limit HCV infection in Europe.

## Abbreviations

DAA: Direct-acting antiviral
GBD: Global burden diseases project
HCV: Hepatitis C virus
IU/ml: International units per Milliliter
IVDA: Intravenous drug abuser
MSM: Man who have sex with men
PWID: People who inject drugs
RT-PCR: Reverse transcription polymerase chain reaction
